# Bio-inspired low-tortuosity carbon host for high-performance lithium-metal anode

**DOI:** 10.1093/nsr/nwy148

**Published:** 2018-11-26

**Authors:** Yi-Chen Yin, Zhi-Long Yu, Zhi-Yuan Ma, Tian-Wen Zhang, Yu-Yang Lu, Tao Ma, Fei Zhou, Hong-Bin Yao, Shu-Hong Yu

**Affiliations:** 1 Department of Chemistry, CAS Center for Excellence in Nanoscience, Hefei Science Center of CAS, University of Science and Technology of China, Hefei 230026, China; 2 Hefei National Laboratory for Physical Sciences at the Microscale, University of Science and Technology of China, Hefei 230026, China; 3 CAS Key Laboratory of Mechanical Behavior and Design of Materials, Department of Modern Mechanics, University of Science and Technology of China, Hefei 230026, China

**Keywords:** bio-inspiration, low-tortuosity carbon host, lithium metal, micro-channels, nucleation sites

## Abstract

Lithium metal is one of the most promising anode materials for high-energy-density Li batteries. However, low stability caused by dendrite growth and volume change during cycling hinders its practical application. Herein, we report an ingenious design of bio-inspired low-tortuosity carbon with tunable vertical micro-channels to be used as a host to incorporate nanosized Sn/Ni alloy nucleation sites, which can guide Li metal's plating/stripping and meanwhile accommodate the volume change. The pore sizes of the vertical channels of the carbon host can be regulated to investigate the structure–performance correlation. After compositing Li, the bio-inspired carbon host with the smallest pore size (∼14 μm) of vertical channels exhibits the lowest overpotential (∼18 mV at 1 mA cm^−2^), most stable tripping/plating voltage profiles, and best cycling stability (up to 500 cycles) in symmetrical cells. Notably, the carbon/Li composite anode is more rewarding than Li foil when coupled with LiFePO_4_ in full cells, exhibiting a much lower polarization effect, better rate capability and higher capacity retention (90.6% after 120 cycles). This novel bio-inspired design of a low-tortuosity carbon host with nanoalloy coatings may open a new avenue for fabricating advanced Li-metal batteries with high performance.

## INTRODUCTION

The ever-increasing demand for high-energy-density storage devices is pushing ahead fundamental studies on high-specific-capacity electrode materials [1–6]. Lithium metal, as one of the most promising candidates for the next-generation anode materials in high-energy-density Li batteries, possesses the highest theoretical specific capacity (3860 mAh g^−1^) and the lowest electrochemical potential (−3.04 V vs. a standard hydrogen electrode) [7–10]. However, dendritic Li growth, continuous electrolyte consumption, and huge volume change during battery cycling become the ‘Achilles’ heel’ of Li-metal anode, causing serious safety hazards, low Coulombic efficiency and deterioration of the whole electrode [11–16]. As a result, the utilization of Li-metal anodes in high-energy-density rechargeable Li-metal-based batteries has long been hindered.

Over the past few decades, several strategies derived from different aspects of materials design have been proposed to address the above-mentioned issues of Li-metal anode. One of these methods is to stabilize or modify a solid electrolyte interphase (SEI) on the surface of the Li-metal anode by introducing some certain additives to the liquid electrolyte [17]. The identical point shared by these additives is that they can interact actively with Li metal to form a protective layer on the surface of the Li-metal anode [18–21]. However, the idea of reinforcing SEI seems a little bit powerless to restrain the huge volume change of Li-metal anode during the Li stripping/plating process, which tends to iteratively extrude the SEI layer and has a tremendous potential to cause cracks [22]. Once the cracks occur, fresh Li metal underneath is exposed to the electrolyte, which will induce its further reaction with the electrolyte and inhomogeneous deposition, causing low Coulombic efficiency and Li dendritic growth.

Recently, an alternative strategy to accommodate the huge volume change of Li-metal anode has been proposed by using 3D host scaffolds [23]. The pioneering work done by Cui *et al*. manifested the use of a 3D porous framework with a ‘lithiophilic’ surface after Si coating to fabricate porous scaffold-hosted Li-metal anode with excellent structural stability upon galvanostatic cycling [24]. Similarly, to build up efficient hosts for accommodating Li metal in anodes, various hierarchically 3D structural designs have been proposed including graphene layers [16], a polymeric matrix [25], a porous Cu current collector [26], and several other porous hosts [27–29]. However, these reported porous hosts were constructed by random networks without considering the ordered structural design and nucleation site guidance to improve the efficiencies of hosts for the plating and stripping of Li.

Recently, Yet-Ming Chiang *et al.* and our group have demonstrated that a low-tortuosity structural design for hosts could enhance the performance of electrodes without changing the underlying material chemistry [30,31]. For the Li-metal anode, interestingly, although the micro-channeled carbon hosts with low tortuosity have been prepared by using natural woods as templates [32,33], the pore sizes of the micro-channels in natural woods are hard to control because they are related to many environmental factors including sunlight, temperature, age of the tree and sampling position [34]. In addition, the nucleation sites for guiding the plating of Li inside the vertical channels have not been thoroughly considered as well. Therefore, the structural consistency of as-fabricated Li metal composite anode is hard to maintain for investigating the correlation between structure and performance to further optimize the electrode design.

Herein, to comprehensively study the low-tortuosity host for Li-metal anode, a unidirectional ice-templating technique was adopted to fabricate carbon hosts with size-tunable vertical channels [35] inspired by wood structures and Sn/Ni alloy nanoparticle coatings inside, in which molten Li was infused to form low-tortuosity Li metal composite anode. By controlling the initial temperature (*T*_i_) of the unidirectional freezing procedure, a series of carbon hosts with vertical channels (CHVC) of different pore sizes are constructed. For the sake of convenience, a sample of CHVC with a *T*_i_ of *T*°C is denoted by CHVC-*T* (e.g. a sample with an initial temperature of −30°C is denoted by CHVC-30). After electroplating Sn/Ni nanoparticles into the as-fabricated CHVC, the inner surfaces of the vertical channels become ‘lithiophilic’, which facilitates the infusion of molten Li into the CHVC to form CHVC/Li composite anodes. Notably, the Sn/Ni nanoparticle coating on the inner surface of the CHVC could induce homogeneous nucleation and plating of Li during battery cycling [36], which significantly lowers the local current density and improves the cycling stability of the CHVC/Li composite anode. We demonstrated that when the channel size of CHVC decreased to 14 μm by setting *T*_i_ to −30°C, CHVC-30/Li composite electrodes can achieve the lowest overpotential of 20 mV up to 500 cycles in a symmetric cell (1 mA cm^−2^, 1 mAh cm^−2^). Furthermore, the CHVC-30/Li-LiFePO_4_ full cell also exhibited a lower polarization effect (205 mV) at high rate (5 C) and a high capacity retention of 90.6% at 1 C after 120 cycles.

## RESULTS AND DISCUSSION

### Construction of tunable vertical channels in the carbon host

**Figure 1.**
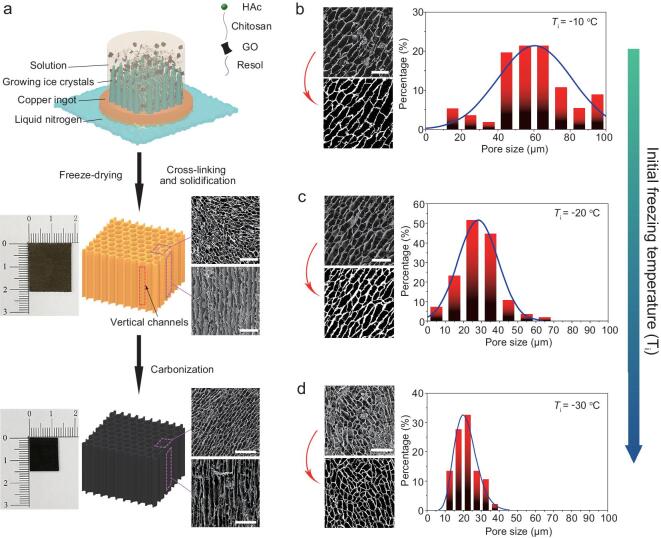
Schematic of a typical fabrication process of CHVC and size-controlling of vertical channels in PMVC via different *T*_i_. (a) Schematic image of the solution used for directional freezing (top), real picture, top-view and side-view SEM images of PMVC (middle) and CHVC (bottom). (b, c, d) Original and processed top-view SEM images of PMVC prepared via *T*_i_ of −10°C (b), −20°C (c) and −30°C (d). The corresponding statistical histograms of the pore size indicate the good controllability of the channel sizes via different *T*_i_ (all scale bars: 100 μm).

We adopted a unidirectional freezing, freeze-drying, polymer solidification, and carbonization process to construct the carbon hosts with tunable vertical channels. As shown in Fig. 1a, a polymer matrix with vertical channels (PMVC) was first constructed via the ice-induced self-assembly and thermocuring process [35], and then the obtained PMVC was carbonized in Ar atmosphere to obtain a carbon host with vertical channels (CHVC). At first, chitosan, acetic acid, resol and graphene oxide nanosheets were dispersed into deionized water to form a precursor suspension for directional freezing (see the section entitled ‘Methods’). The top part of Fig. 1a illustrates the formation of vertical channels induced by directional growth of ice crystals during the freezing process. After the freeze-drying processes, a microstructure with vertical channels in a cryogel matrix was preliminarily formed. To stabilize this structure, the sample was solidified and cross-linked at 180°C for 1.5 h to obtain the PMVC (middle part of Fig. 1a). The bulk size of the as-obtained PMVC can be up to 4 cm × 4 cm × 3 cm, which is easy to cut into regular slices with dimensions of 21 mm × 18 mm × 0.7 mm (Supplementary Fig. 1). In addition, the obtained PMVC retained vertically aligned micro-channels inside the polymer matrix (see the scanning electron microscopy (SEM) images in the middle part of Fig. 1a). Finally, the fabricated PMVC framework was carbonized in Ar/H_2_ atmosphere at 900°C for 6 h to generate the CHVC. As shown in the bottom part of Fig. 1a, after the carbonization, although the size of the slice reduced to 15 mm × 13 mm × 0.5 mm, the integrity of the carbonized framework was maintained very well with vertical channels throughout the whole CHVC (see the SEM images in the bottom part of Fig. 1a). The process of carbonization resulted in a shrinkage of 31% in the sample's macroscopic size, which is consistent with the reduction of pore size in the vertical channels (Supplementary Table 1). Notably, the advantage of our developed method to fabricate PMVC is that the pore sizes of vertical micro-channels generated in PMVC can be tuned via different initial freezing temperatures (*T*_i_) (Fig. 1b, c and d). We compared the pore-size distribution of vertical channels in CHVC based on the analysis of SEM images as shown in Fig. 1b–d. During the statistics of channel sizes, a thresholding method supplemented with an artificial image inpainting process was applied to eliminate the adverse effects caused by the slice cutting, such as fragments and wall breakage (Supplementary Fig. 2a, as denoted by the red arrows). Furthermore, the shortest diameter via the geometric center was defined as the pore size (Supplementary Fig. 2b and c). As *T*_i_ decreased from −10°C to −20°C and −30°C, the average pore size based on the distribution statistics of PMVC presented an obvious shift from 60 μm to 30 μm and 20 μm, respectively. This phenomenon can be explained by the relationship between the degree of supercooling and nucleation of water in the freezing precursor solution. When *T*_i_ is reduced, the degree of supercooling increased, which leads to the formation of more crystal nuclei of ice. In contrast, at a relatively higher temperature below 0°C, the ice crystal nuclei have enough time to grow, which results in a larger pore size in the channels. The shapes and sizes of the vertical channels in PMVC are inherited from ice crystals; therefore, thinner ice crystals result in a smaller pore size in the channels, which finally determines the pore size of the vertical channels in CHVC.

### Fabrication of CHVC/Li composite anode via Li infusion into CHVC

**Figure 2.**
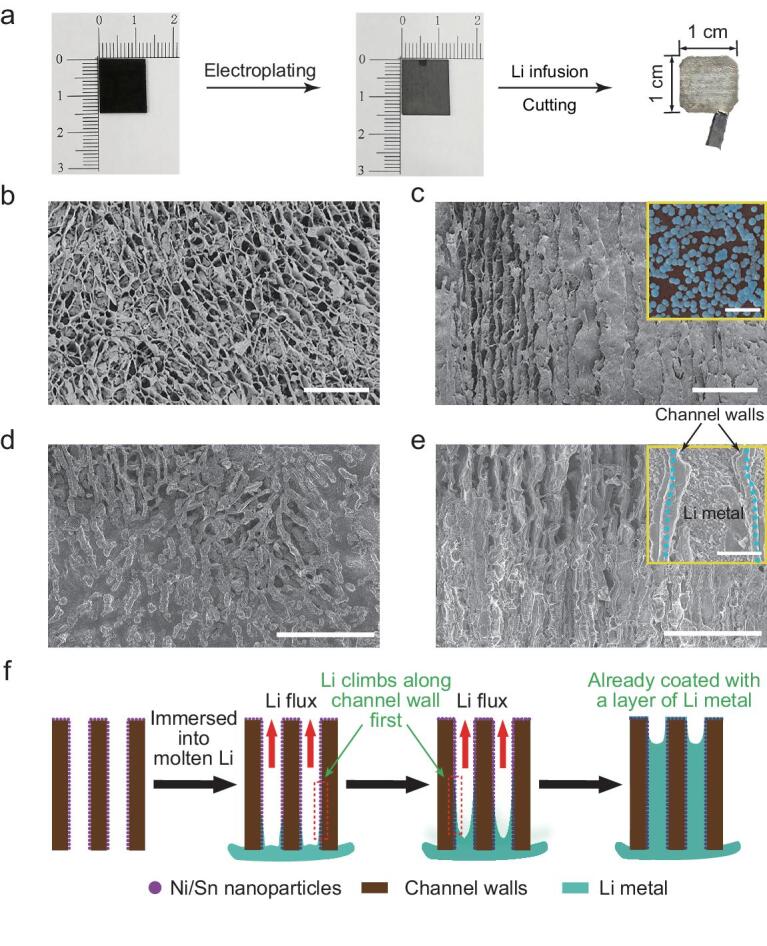
Schematic of the fabrication of the CHVC/Li composite. (a) Photographs of the fabricated CHVC (left), Sn/Ni-coated CHVC (middle) and CHVC/Li composite (right). (b) Top-view SEM image of Sn/Ni-coated CHVC (scale bar, 100 μm). (c) Side-view SEM image of Sn/Ni-coated CHVC (scale bar, 100 μm) and a partially enlarged view (scale bar, 1 μm) of the channel walls of Sn/Ni-coated CHVC (inset). (d) Top-view SEM image of CHVC/Li composite (scale bar, 100 μm), the channel walls of which can still be observed, indicating the integrity of the structure after Li infusion. (e) Side-view SEM image of CHVC/Li composite (scale bar, 100 μm) and a partially enlarged view of the vertical channel longitudinal sections (scale bar, 10 μm), displaying the morphology of the Li metal and channel walls after Li infusion. (f) Schematic diagrams of a typical process of Li infusion into Sn/Ni-coated CHVC matrix inferred from the above characterizations.

The weak affinity between carbon and lithium makes it very hard to infuse molten Li into the porous CHVC. The electrochemical deposition method was adopted to coat the inner surface of the micro-channels in the CHVC with Sn/Ni nanoparticles. As shown in Fig. 2a, after electroplating, the color of CHVC changed from black to gray. The multiscale characterization of Sn/Ni nanoparticle-coated CHVC shows that the nanoparticles dispersed homogeneously on the whole inner surface of CHVC (Supplementary Figs 3, 12 and 13) and the open vertical channels in the CHVC are still maintained (Fig. 2b and c). In addition, the partially enlarged view of the surface of the channel walls in Fig. 2c shows that Sn/Ni alloy nanoparticles possess the geometric profile of a sphere and are almost the same size (about 150 nm in diameter). The spherical geometric profile and homogeneous size of Sn/Ni nanoparticles can be attributed to the utilization of a high-voltage pre-nucleation technique (HVPNT) during the electroplating process (see the section entitled ‘Methods’, Supplementary Fig. 4 and Supplementary Note 2). Supplementary Fig. 4a exhibits the morphology of nanoparticles electroplated with the application of HVPNT; they are uniform in size and homogeneous in distribution. In comparison, the Sn/Ni electroplating without applying HVPNT resulted in irregular nanoparticles with an obviously random distribution of shape and size (Supplementary Fig. 4b). Moreover, the phase of Sn/Ni nanoparticles electroplated onto the inner surface of CHVC was confirmed by powder X-ray diffraction (PXRD) as Ni_3_Sn_2_ (Supplementary Fig. 4c).

As expected, after immersing the Sn/Ni-coated CHVC slice in molten Li, the Li smoothly flowed into CHVC due to the good wettability and capillary forces and then shiny silvery CHVC/Li composite anode was obtained (Supplementary Movie 1 and right image of Fig. 2a). After Li infusion, the fact that the channel walls can still be observed in the top-view SEM image (Fig. 2d) and cross-sectional-view SEM image (Fig. 2e) of the CHVC/Li composite slice further confirmed the integrity of the structure with vertical channels in the CHVC after Li infusion.

To investigate the process of Li infusion into vertical channels with the help of the Sn/Ni alloyed nanoparticle coatings, a sample of a CHVC/Li composite slice with part infusion of molten Li was comprehensively characterized by SEM. From the top-view SEM image of partly infused CHVC/Li composite, we can see a lot of protrusions, which can be attributed to the emergence of channel walls caused by volume contraction of Li upon cooling (Supplementary Fig. 5a). As we can see from the enlarged top-view SEM image in Supplementary Fig. 5b, the surfaces of the channel walls are covered with Li metal, no matter whether the walls belonged to channels with half Li infusion (encircled by a green dotted line) or full Li infusion (encircled by a blue dotted line). The contrast between channels with half Li infusion and channels with full Li infusion indicates a more detailed process of Li infiltration in which Li covers the wall surface prior to its filling into the center void space of the surrounding channel walls. Supplementary Fig. 5c shows a side-view SEM image of the CHVC/Li slice, which indicates that part of the void space in the channels is occupied by Li. As shown in the enlarged SEM image (Supplementary Fig. 5d), the channel walls can be clearly observed, confirming the integrity of the vertical channels of the matrix after Li infiltration. The general process of Li infusion inferred from the above discussion is illustrated in Fig. 2f. After immersing Sn/Ni-coated CHVC in molten Li, the molten Li first climbs along the channel walls due to the lithiophilic particle coatings, followed by Li filling into the middle void space in channels to form the CHVC/Li composite anode.

### Electrochemical performance of CHVC/Li composite anode

To evaluate the electrochemical performance of CHVC/Li composite anode with different pre-designed vertical channel sizes, symmetrical coin cells (2032-type) with two identical CHVC/Li composite electrodes were assembled by using 1.0 M LiTFSI, 1 wt% LiNO_3_ in 1,3-dioxolane/1,2-dimethoxyethane (DME:DOL = 1:1 Vol%) as the electrolyte. Meanwhile, control cells were fabricated by using two bare Li foils as electrodes with the same thickness (∼500 μm). The cells with CHVC-10, CHVC-20, CHVC-30 or bare Li as electrodes were cycled with an areal capacity of 1 mAh cm^−2^ under a current density of 1 mA cm^−2^.

**Figure 3.**
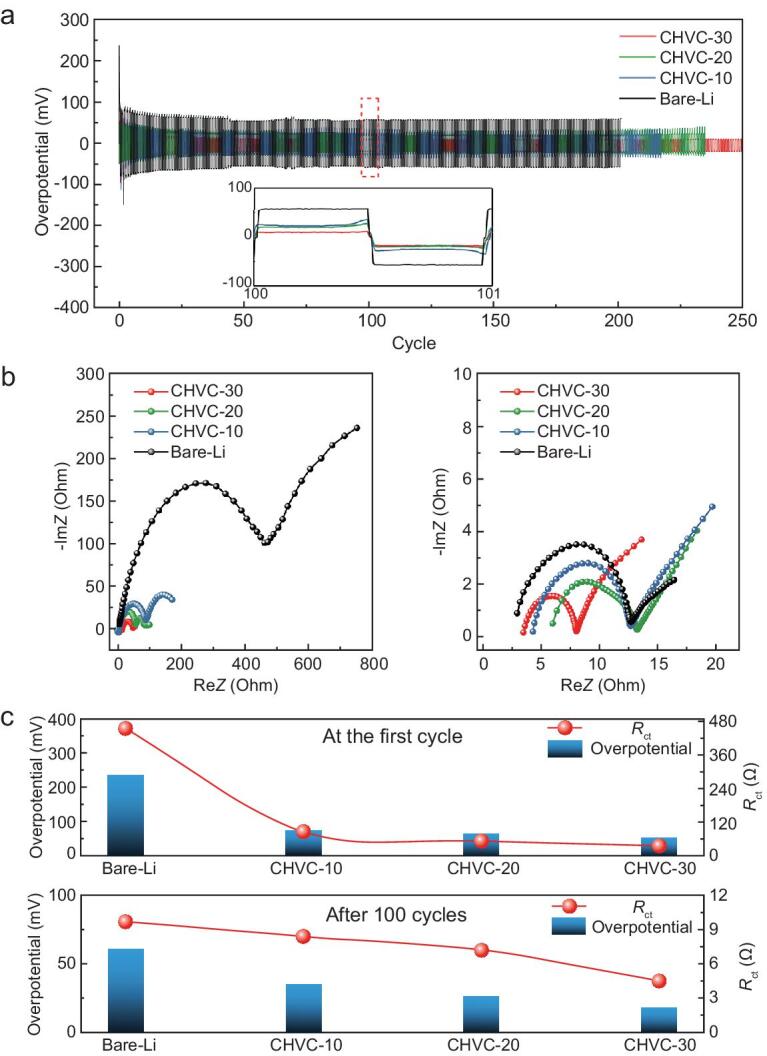
Electrochemical characterization of bare Li anode and CHVC-*T*_i_/Li composite anode. (a) Cycling performance of symmetric cells with CHVC-30/Li, CHVC-20/Li, CHVC-10/Li and bare Li at a current density of 1 mA cm^−2^. (b) Nyquist plot of the impedance spectra of symmetric cells with CHVC-30/Li, CHVC-20/Li, CHVC-10/Li and bare Li before cycling and after 100 cycles. (c) Variation trends of *R*_ct_ values and overpotential among symmetric cells with CHVC-30/Li, CHVC-20/Li, CHVC-10/Li and bare Li.

As shown in Fig. 3a, cells with CHVC-10, CHVC-20 and CHVC-30 exhibit a stable voltage profile over long-term cycling compared to the cell with Li foils. Interestingly, the overpotentials of cells using CHVC/Li composite electrodes decreased in the order of CHVC-10, CHVC-20 and CHVC-30, indicating that a smaller pore size in the vertical channels leads to a lower overpotential. In particular, at the first cycle, the overpotentials of CHVC-10, CHVC-20, CHVC-30 and Li foil are 52 mV, 65 mV, 74 mV, and 237 mV, respectively. Furthermore, after 100 cycles, the overpotentials of the different electrodes in the above order are 18 mV, 26 mV, 35 mV, and 61 mV, respectively (Fig. 3a, insets). Obviously, CHVC/Li composite electrodes exhibit superior cycling stability in comparison to that of bare Li foil. This improved cycling performance can be attributed to two aspects. On one hand, vertical channels in CHVC, as the host for Li, can accommodate the undesired volume change during cycling, which improves the integrity of the electrode. On the other hand, the low tortuosity and Sn/Ni alloy nanoparticle coating endows the matrix with a high specific surface area, lowering the practical areal current density, shortening the Li-ion transport path and enabling uniform Li nucleation and growth upon Li plating due to the more homogeneous contact with Li^+^-ion flux (Supplementary Fig. 6 and Supplementary Note 3). Furthermore, an alternating current (AC) impedance test was performed to provide more evidence of low polarization in CHVC/Li composite electrodes. As we can see from the electrochemical impedance spectra (EIS) in Fig. 3b, before cycling (left graph), the charge transfer resistance (*R*_ct_) values of symmetric cells with CHVC/Li composite electrodes are all less than 100 Ω, which is much lower than that of the cell with bare Li-foil electrodes (*R*_ct_ more than 450 Ω). In particular, CHVC-30/Li exhibits the lowest *R*_ct_ of only 34 Ω. After 100 cycles, compared to the cell with bare Li-foil electrodes (*R*_ct_ 10 Ω), cells using the CHVC/Li composite anode all presented *R*_ct_ values less than 9 Ω (right graph in Fig. 3b). Notably, among cells with CHVC/Li composite electrodes, the values of *R*_ct_ are also relevant to the sizes of vertical channels, which is in accordance with the variation trend of overpotential, as shown in Fig. 3c. We can infer from these results that a porous matrix with vertical channels enables a faster charge transfer and a quicker kinetics of electrochemical reaction with less resistance, which can be attributed to an enlarged contact area between the Li metal and electrolyte (Supplementary Fig. 7). Therefore, cells using CHVC/Li composite electrodes, especially those with smaller pore sizes, will display a significantly improved cycling performance, the conclusion of which is consistent with a previous report about a porous copper current collector [37]. In fact, symmetric cells with CHVC-30/Li composite electrodes can maintain their low overpotential (18 mV) and stable voltage profile up to 500 cycles (Supplementary Fig. 8). Because CHVC-30/Li showed the best electrochemical performance in the symmetric cell test, it was used for further characterizations and electrochemical performance tests.

### Structural integrity of CHVC-30/Li composite anode during Li stripping

**Figure 4.**
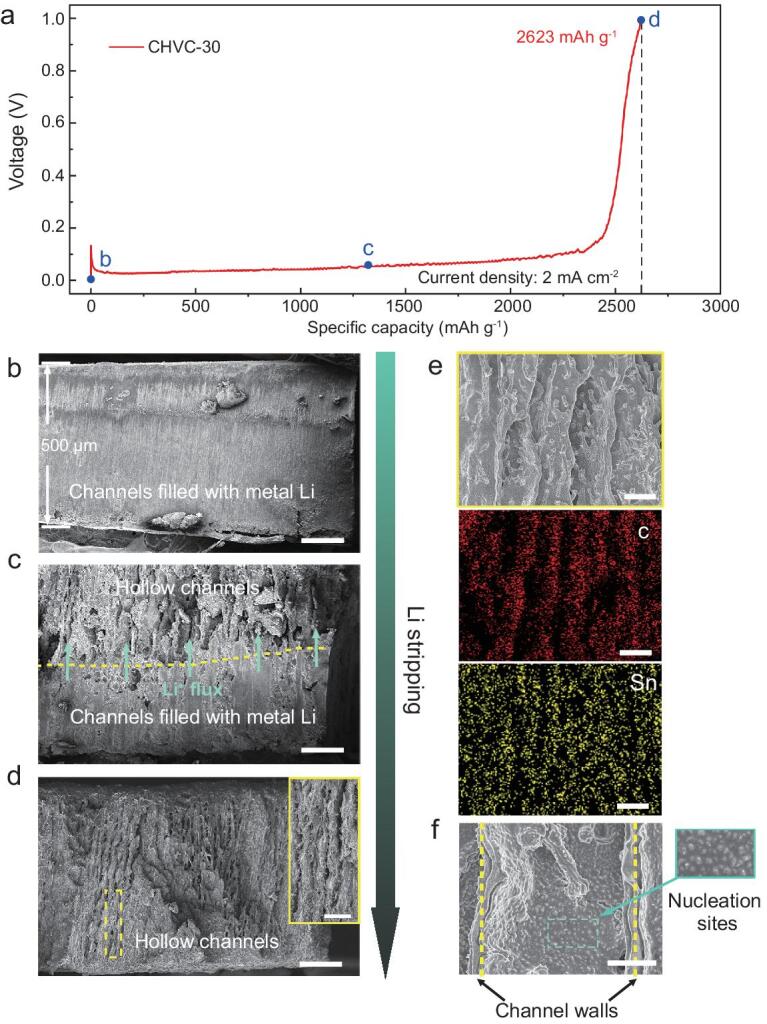
Exploration of the CHVC-30/Li composite electrode's capacity and the structural evolution of the electrode during the Li stripping process. (a) Typical electrochemical curve of Li stripping for CHVC-30/Li composite electrode at a current density of 2 mA cm^−2^, which delivers a specific capacity of 2623 mAh g^−1^ when the cut-off voltage is set at 1 V. (b–d) A side-sectional SEM image of CHVC-30/Li composite electrode before Li stripping (marked as ‘**b**’ in (a)) and when 1300 mAh g^−1^ (about half of the total capacity, marked as ‘**c**’ in (a)) of Li and all of Li (∼2623 mAh g^−1^, marked as ‘**d**’ in (a)) has been stripped out (scale bars, 100 μm). The partially enlarged image (scale bar, 30 μm) in (d) reveals the well preserved structure after the Li stripping process. (e) A side-sectional SEM image of the vertical channels in CHVC-30/Li composite electrode after Li stripping (top) and corresponding elemental maps from EDX analysis showing the elemental distributions of C (middle) and Sn (bottom). (f) SEM image of the inner surface of a single channel, on which many nucleation sites can be clearly observed.

The long-term cycling test above has confirmed the sufficient strength of channel walls in CHVC-30/Li composite electrodes to accommodate the volume change of Li metal. The structural integrity, however, also has the potential to be threatened by the pressure caused by battery cases, which is needed to ensure that batteries are properly leakproof. Our carbon host with Li metal inside can easily withstand the pressure from battery cases due to the support of the soft Li metal inside. However, if a structure with vertical micro-channels can still maintain its integrity after all the Li metal inside has been stripped out, the structural integrity during and after electrochemical processes can be further confirmed. Thus a Li-stripping test was conducted after 100 cycles with a cycling capacity of 5 mAh cm^−2^ to investigate the structural integrity, as well as the specific capacity of CHVC-30/Li composite electrodes. The specific capacity of CHVC-30/Li composite anode was tested with symmetric cells at a current density of 2 mA cm^−2^. As shown in Fig. 4a, CHVC-30/Li composite electrode can deliver a specific capacity of 2623 mAh g^−1^, which is two-thirds of the theoretical specific capacity of Li metal (3860 mAh g^−1^). To illustrate the structural evolution of CHVC-30/Li composite anode during the stripping process, SEM was performed on the cross-sectional area before stripping (Fig. 4b), after Li stripping of 1300 mAh g^−1^ (about half of the total capacity, Fig. 4c), and when the stripping process was finished (Fig. 4d). Compared with Fig. 4b, which presents the morphology of the CHVC/Li composite electrode before the Li stripping process, Fig. 4c displays a clear boundary (yellow dotted line) dividing the channel space with and without Li stripping. The boundary line lies in the middle of the cross-section of the CHVC/Li composite anode, which is consistent with the proportion of the amount of already-stripped Li to the total capacity. As the the stripping process goes on, the Li^+^-ion flux continues flowing into the electrolyte from the Li metal, causing the boundary line to move downward (as denoted by cyan arrows in Fig. 4c). When the capacity of the stripped Li reaches 2623 mAh g^−1^, all vertical channels become almost hollow (Fig. 4d). The profiles of the vertical channels can still be observed after all the Li has been stripped out, revealing that the structure of the CHVC matrix is well maintained during and after the Li stripping process. In addition, when the cell was disassembled after the stripping process had finished, it was observed that the shape of the CHVC-30/Li composite electrode was still as well preserved as that of the electrode before testing, with only the color changing from silver to black due to the stripping-out of Li (Supplementary Fig. 9). Thus both macroscopic and microscopic observations confirmed the structural integrity of the CHVC matrix during the Li stripping process.

Interestingly, after Li stripping, small-sized protrusions were found to be densely dispersed on the inner surface of the micro-channels (Fig. 4e, top). The corresponding elementary mappings with energy dispersive spectroscopy (EDS) show that the carbon mapping pattern is similar to the shape of the micro-channels and the tin signal homogeneously appears in the micro-channels (Fig. 4e, middle and bottom), which indicates that the densely dispersed protrusions are tin-alloyed nanoparticles originating from the pristine Ni/Sn nanoparticles. Furthermore, the enlarged SEM image of an individual channel in Fig. 4f reveals an extremely rough surface endowed with protrusions, which largely dissipates the current density of lithium plating. Therefore, the densely dispersed tin-alloyed nanoparticles left in the micro-channels after Li stripping could act as nucleation sites, inducing homogeneous Li plating in the carbon host for the following cycles.

### Full cell performance of CHVC-30/Li anode coupled with LiFePO_4_ cathode

**Figure 5.**
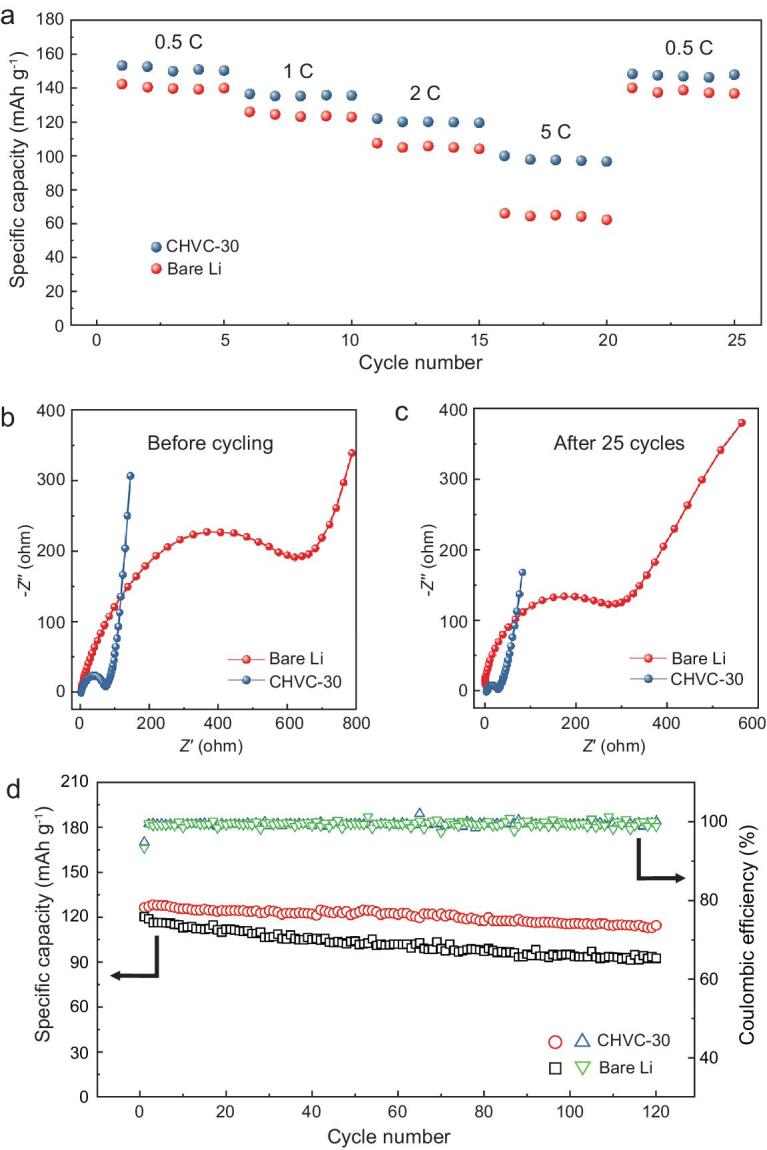
Electrochemical performance of full cells with LiFePO_4_ as cathode versus bare Li or CHVC-30/Li composite as anode. (a) Rate performance of two cells. (b, c) The comparison of impedance spectroscopy of two cells before cycling (b) and after 50 cycles (c). (d) Cycling performance of two cells at 1 C.

To prove the advantage of using CHVC-30/Li composite anode to improve the performance of Li-metal-based batteries, full cells with LiFePO_4_ (LFP) as cathodes versus CHVC-30/Li composite anodes or bare Li foil were assembled and tested. Fig. 5a shows a comparison of the rate capacity of full cells with bare Li foil or CHVC-30/Li composite anodes. At low rates below or equal to 1 C, the specific capacities of two cells are relatively close to each other (i.e. the specific capacity is 151 mAh g^−1^ for CHVC-30/Li composite anode and 140 mAh g^−1^ for bare Li foil at 0.5 C). As the rate increases to 2 C or higher, however, the differences between the two cells using different anodes become more obvious. At the rate of 2 C, the value difference between the capacities of the two cells reaches 15.3 mAh g^−1^. In particular, the cell assembled with CHVC-30/Li composite anode presents a specific capacity of 97 mAh g^−1^ at 5 C, while that of the Li-foil/LFP full cell is only 62 mAh g^−1^. Supplementary Fig.10a and b displays the voltage profiles of the two cells at different rates. As we can see, cells with CHVC-30/Li composite anode exhibit a much lower polarization effect (205 mV at 5 C, Supplementary Fig. 10a) than that of the bare Li/LFP full cell (600 mV at 5C, Supplementary fig. 10b). To further confirm the low polarization effect endowed by the vertical channels of CHVC-30/Li anode, impedance spectra were performed before cycling and after 25 cycles of the above-mentioned rate capacity test (Fig. 5b and c). The charge transfer resistance of the cell with CHVC-30/Li composite anode is 83 Ω before cycling, about one-eighth of that of the Li-foil/LFP full cell (>600 Ω). Similarly, after the rate capability test, compared to the full cell using bare Li-foil anode (*R*_ct_ 270 Ω), the cell using CHVC-30/Li composite anode exhibits a much smaller *R*_ct_ of 40 Ω after the rate capability test. When the two cells were tested at 1 C for 120 cycles, a capacity retention rate of 90.6% was realized by the cell with CHVC-30/Li composite anode, while that of the cell with bare Li was only 82.6% (Supplementary Table 2). The better electrochemical performance of CHVC-30/Li composite anode in full cells indicates the effectiveness of introducing micro-channels into Li-metal anodes to broaden the application areas of Li-metal batteries.

The ice-templated CHVC is similar, but superior, to a natural wood-templated framework because it is size-tunable and possesses a more homogeneous pore-size distribution (Fig. 1b–d). On the basis of the pore-size tunability of CHVC, we could further investigate the correlation between the pore size in the vertical channels and the electrochemical performance of the as-obtained CHVC/Li composite anode. A smaller pore size leads to better electrochemical performance, which is consistent with the pore-size design in the porous copper current collector [37]. As the result, the CHVC-30/Li composite anode has the lowest overpotential (∼18 mV) and very flat voltage profiles even when it is cycled for 500 cycles (Supplementary Fig. 8).

The excellent electrochemical performance of the as-fabricated CHVC-30/Li composite anode can be attributed to two aspects. First, the contact interface between the Li metal and the electrolyte is a curved surface rather than a flat surface (Supplementary Fig. 7). Compared with flat bare Li foil, this type of contact between Li and the electrolyte enlarges the effective surface area of the Li metal and ensures better Li-ion transfer. As a result, the increased surface area lowers the practical current density, as well as the charge transfer resistance. Thus, the overpotential of CHVC/Li composite electrodes is much lower than that of bare Li foil (Fig. 3a). Furthermore, with a lower practical current density, the nucleation and growth of Li will be uniform and dense, in comparison to the non-uniform and loose morphologies of Li metal plated on the surface of bare Li foil (Supplementary Fig. 6), which causes the surface pulverization of lithium foil. On the other hand, the vertical channels in CHVC can effectively accommodate the volume changes of the anode during the Li stripping/plating process, which largely enhances the stability and integrity of the whole cell. In detail, the Sn/Ni nanoparticles decorated on the carbon walls of the vertical channels, as the nucleation sites for Li plating, can confine the Li stripping/plating to only along the vertical channels. Also, the vertical channels are strong enough to maintain the micro-structure of CHVC during cycling, which is confirmed by our observation of the well preserved channel structures after Li stripping tests (Fig. 4). In addition, in macroscale observation, the CHVC electrode still maintains its original shape after stripping all of the Li metal in the host (Supplementary Fig. 9b).

## CONCLUSION

In summary, we propose a bio-inspired design strategy to construct a low-tortuosity carbon with tunable vertical channels as the efficient host for Li-metal anode to improve the cycling stability and rate performance. With a simple electroplating method, we manage to adhere a coating of Sn/Ni alloy nanoparticles with great homogeneity in size and dispersion onto the inner surface of the micro-channels (Supplementary Figs. 12 and 13). The Sn/Ni alloy nanoparticle-coated vertical channels in the host could guide the uniform nucleation and growth of Li metal to accommodate the volume change and suppress the dendritic growth of Li metal. After compositing Li, the bio-inspired carbon host with the smallest pore size (∼14 μm) of vertical channels exhibits the lowest overpotential (∼18 mV at 1 mA cm^-2^), most stable tripping/plating voltage profiles, and best cycling stability (up to 500 cycles) in symmetrical cells. The vertical micro-channels and the Sn/Ni alloy nanoparticles have a synergistic effect on the facilitation of a uniform nucleation process, lowering the overpotential and improving the stability at high current density (Supplementary Figs. 14 and 15). Attractively, the full cell of LiFePO_4_–CHVC-30/Li exhibits low charge transfer resistance and high rate performance resulting from the vertical channes design in the composite anode. Our proposed Li-metal composite anodes will boost the applications of Li-metal-based high-energy-density batteries via the introduction of 3D microstructure with vertical channels.

## METHODS

### Fabrication of CHVC/Li composite anode

The PMVC was fabricated by a modified ice-induced self-assembly and thermal curing process according to our previous report [35]. Chitosan (2 g) and acetic acid (2 g) were mixed with deionized water (75 mL, 18 MΩ, Q-POD Element) in a beaker sealed with a piece of parafilm to form chitosan hydrosol. Then the chitosan hydrosol was vigorously stirred with a magnetic stirrer for 1 h to form a low-viscosity aqueous dispersion. After that, graphene oxide solution (7 mL, 7.1 mg mL^−1^) was added into the above dispersion, followed by the addition of resol (7.2 g). The resol used in this work was fabricated with formaldehyde, phenol, sodium hydroxide, hydrochloric acid (all from SCRC, AR) and deionized water, according to our previous work [35]. The mixture in the beaker was stirred for another 30 min to make a uniform dispersion. Then the as-prepared solution was poured into a polydimethylsiloxane (PDMS) mold (6 cm × 6 cm × 4.5 cm), which was put on a copper ingot with the pre-cold temperature (−10, −20 or −30°C). The rate of temperature decrease from the copper ingot surface to aqueous dispersion was ∼0.2°C s^−1^, which was controlled via the liquid nitrogen surrounding the copper ingot. After the solution was completely frozen from bottom to top, the solid samples were freeze-dried at −40°C and 10 Pa by a lyophilizer (LABCONCO, FreeZone 2.5 Liter Benchtop Freeze Dryers) for 48 h. Then the obtained cryogel samples were solidified and cross-linked in a chamber furnace (MTI, KSL-1700X) at 180°C for 1.5 h to form PMVC (4 cm × 4 cm × 3 cm). After that, the bulk PMVC was cut into slices with a size of 2.06 cm × 1.82 cm × 0.75 cm by a wire-cutting machine (STX-202A Mini Diamond Wire Saw). Then the slices were carbonized in a tube furnace (MTI, OTF-1200X-5S) in Ar/H_2_ at 900°C for 6 h to form CHVC. Then the generated CHVC slices were coated with Sn/Ni nanoparticles via electroplating. The electroplating solution was prepared by dissolving SnSO_4_ (7.52 g), NiSO_4_.6H_2_O (1.31 g), K_4_P_2_O_7_ (38.44 g), glycine (1.8768 g) and ammonium hydroxide (1 mL) into deionized water (200 mL). The electroplating processes were set as follows (two steps): a relatively high pulse voltage was provided to realize a square-wave pulse current with an alternation of 10 mA cm^−2^ and 0; this was the first step of the electroplating process, namely, the HVPNT. The square-wave pulse current lasted for 200 s, in which the pulse duty ratio was 50% and the frequency was 5 Hz. Then a galvanostatic current of 1 mA cm^−2^ was applied for 2 h; this was the second step of the electroplating process. The electroplated CHVC was washed by deionized water three times and dried in a vacuum oven at 80°C for 6 h. Finally, to infuse Li metal into the micro-channels, the Sn/Ni-coated CHVC was immersed in molten Li (heated on a hot plate of 450°C), as shown in Supplementary Movie 1.

### Material characterization

X-ray diffraction of patterns was carried out on a PW1710 instrument with CuKα radiation (λ = 0.154 06 nm). The microstructure of all the samples was observed by SEM (Zeiss Supra 40) at an acceleration voltage of 5 kV. Elementary analysis was performed with an energy dispersive spectrometer (X-Max^N^, Oxford Instruments).

### Electrochemical measurements

The processes of Li stripping and plating were investigated via symmetric cells (2032-type coin cell) with commercial separators (Celgard 2250). The electrolyte used for testing was 1.0 M LiTFSI, 1 wt% LiNO_3_ in 1,3-dioxolane/1,2-dimethoxyethane (DME:DOL = 1:1 vol%). The LiFePO_4_ cathodes used in the full cell tests were fabricated by blade-coating uniformly mixed slurry (composed of 70 wt% LiFePO_4_, 20 wt% acetylene black and 10 wt% PVDF dispersed in *N*-methyl-2-pyrrolidone (NMP)) onto a piece of aluminum foil followed by vacuum drying for 8 h and cutting into round pieces with diameters of 12 mm to fit the 2032-type coin cells. In rate capacity tests, the charge/discharge rate varied from 0.5 C to 5 C (1 C = 170 mAh g^−1^) under a voltage range of 2.8 V to 3.8 V. The cycling performance of the half cells and rate capacity of full cells were tested with cell-testing equipment (LANHE, CT2001A). EIS measurements were performed by a Biologic VSP-300 multichannel system. All the cells used in our tests were assembled in an argon-filled glove box (O_2_ < 1 ppm, H_2_O < 1 ppm, LABSTAR, LS800S).

## Supplementary Material

Supplemental Files
