# Supplementary material for: Bio-inspired low-tortuosity carbon host for high-performance lithium-metal anode
Source: Natl Sci Rev. 2018 Nov 26;6(2):247–56. doi: 10.1093/nsr/nwy148 (PMC8291544; doi:10.1093/nsr/nwy148)
Supplement: Supplemental Files [file nwy148_supplemental_files.zip › SI_NSR_MS-2018-185.pdf]

Supplementary Information

Bio-inspired low-tortuosity carbon host for high-performance lithium metal anode

Yi-Chen Yin[1★], Zhi-Long Yu[1★], Zhi-Yuan Ma[1], Tian-Wen Zhang[2], Yu-Yang Lu[3], Tao Ma[2], Fei Zhou[2], Hong-Bin Yao[1,2*], Shu-Hong Yu[1,2*]

[1] *Department of Chemistry, CAS Center for Excellence in Nanoscience, Hefei Science Center of CAS, University of Science and Technology of China, Hefei, Anhui 230026, China*
[2] *Hefei National Laboratory for Physical Sciences at the Microscale, University of Science and Technology of China, Hefei, Anhui 230026, China*
[3] *CAS Key Laboratory of Mechanical Behavior and Design of Materials, Department of Modern Mechanics, University of Science and Technology of China, Hefei, Anhui 230026, P. R. China*

★ These authors contributed equally to this work.

* *Correspondence and requests for materials should be addressed to H.B.Y.* (email: yhb@ustc.edu.cn) *or to S.H.Y.* (shyu@ustc.edu.cn)

Supplementary Figures

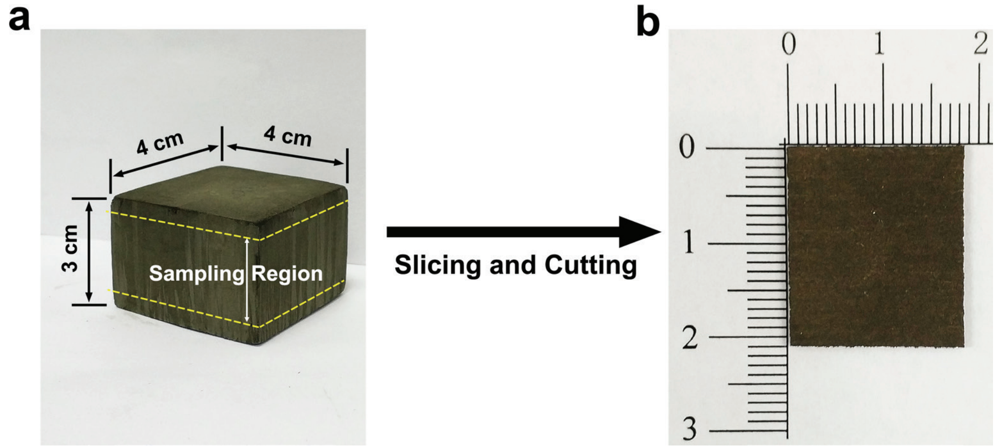

Supplementary Figure 1 | Pictures of an original bulk sample of the as-fabricated PMVC and a slice of that. (**a**) Original bulk sample (4 cm × 4 cm × 3 cm) of PMVC after fabrication processes of directional freezing, freeze drying and solidification. (**b**) A slice sample was cut from the middle part of the PMVC with a diamond-wire cutting machine (*STX-202A Mini Diamond Wire Saw*). The slice size is 21 mm × 18 mm × 0.7 mm. The cut surface is perpendicular to the channel directions.

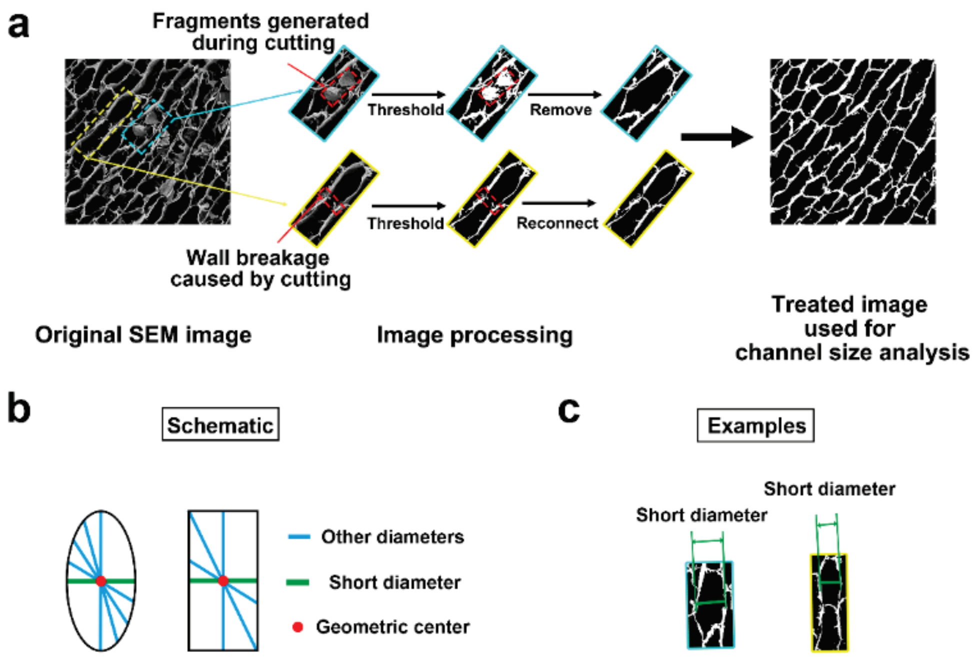

Supplementary Figure 2 | Standardization of SEM image for the statistics of channel size distribution. (**a**) Schematic images of two typical defects caused by cutting that may disturb the statistical count and the restoration processes of the SEM images. (**b**) Graphical representation of the definition of 'short diameter', which was used as the statistical standard for 'pore size' in main text Fig.1 b, c and d. (**c**) Examples of short diameters, namely the 'pore size', of some channels.

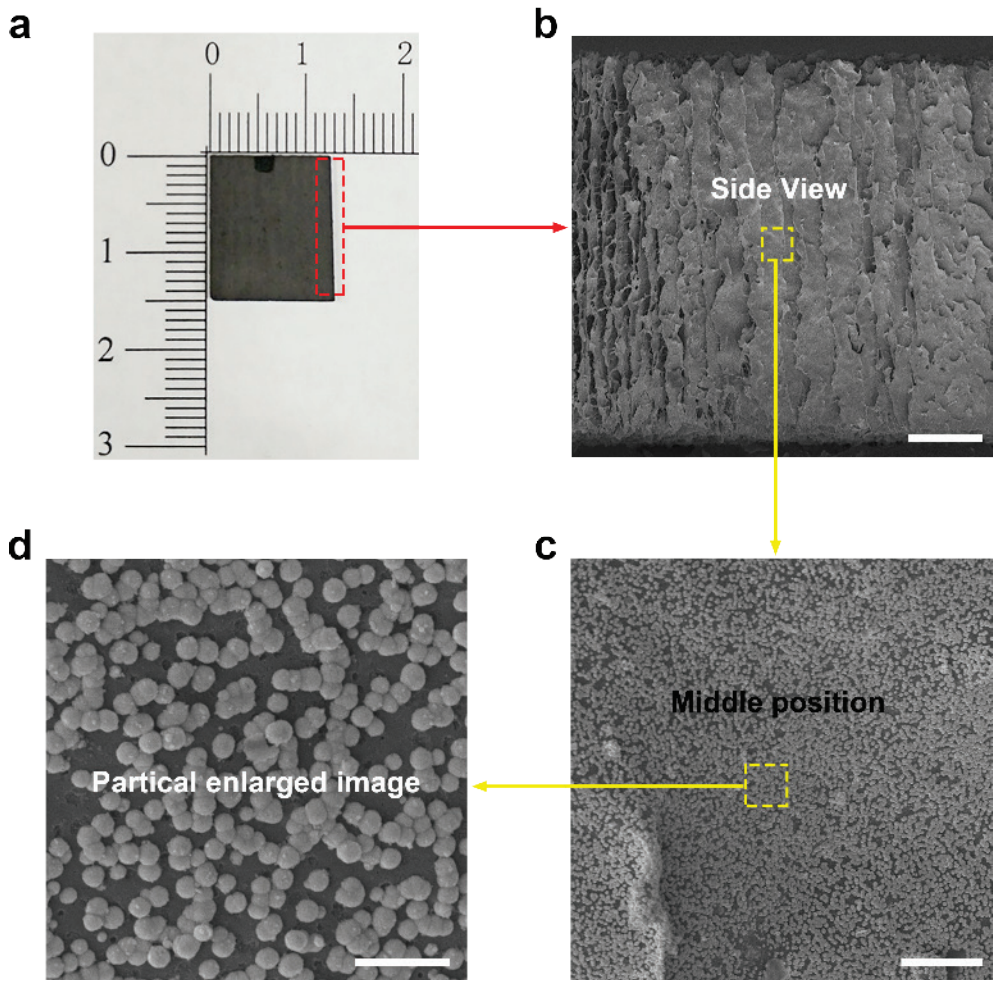

Supplementary Figure 3 | SEM images with different magnification focused on the middle position of the longitudinal section of a Ni/Sn coated CHVC slice, revealing the homogeneous dispersion of Ni/Sn nanoparticles on the inner surface of CHVC even at the deepest area. (**a**) Digital photo of a slice of Ni/Sn coated CHVC, from which we can see the contrast between electroplated area (white area) and nonelectroplated area (the little black square, clamped by a clip during electroplating). (**b-d**) SEM images focused on the middle position of the longitudinal section with magnification times of 100 x (**b**, scale bar, 100 μm), 2500 x (**c**, scale bar, 4 μm) and 25000 x (**d**, scale bar, 500 nm).

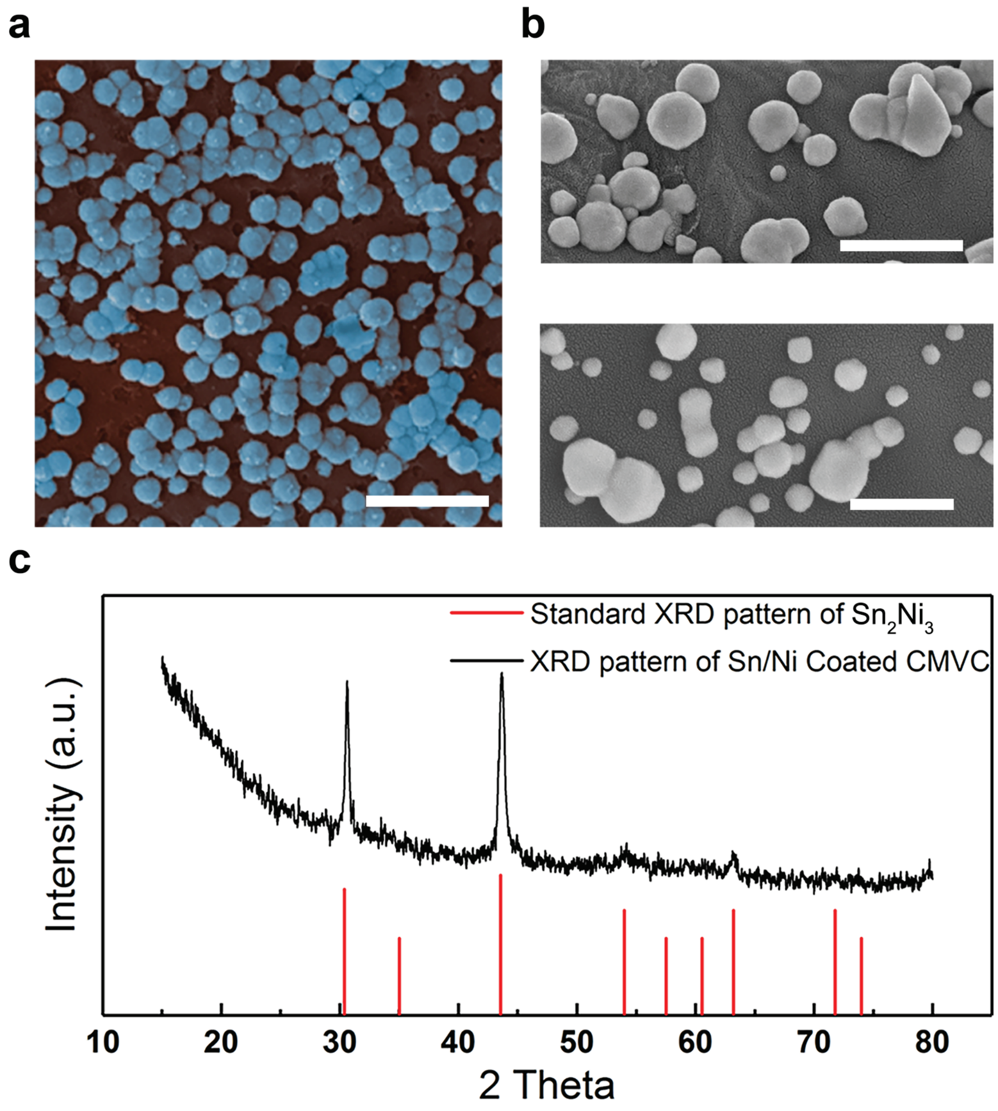

Supplementary Figure 4 | SEM and PXRD Characterizations of electroplated Ni/Sn nanoparticles. (**a**) A SEM image of Ni/Sn nanoparticles electroplated with the application of HVPNT, which shows the homogeneity in size and dispersion of the particles (scale bar, 500 nm, the blue colorization is used to highlight the particles), in comparison to the nonuniform dispersion and size distribution of Ni/Sn paticles without HVPNT (**b**, **top**: scale bar, 500 nm, **bottom**: scale bar, 300 nm). (**c**) PXRD patterns of the electroplated Ni/Sn nanoparticles, revealing the phase of the particles is Sn_2Ni_3.

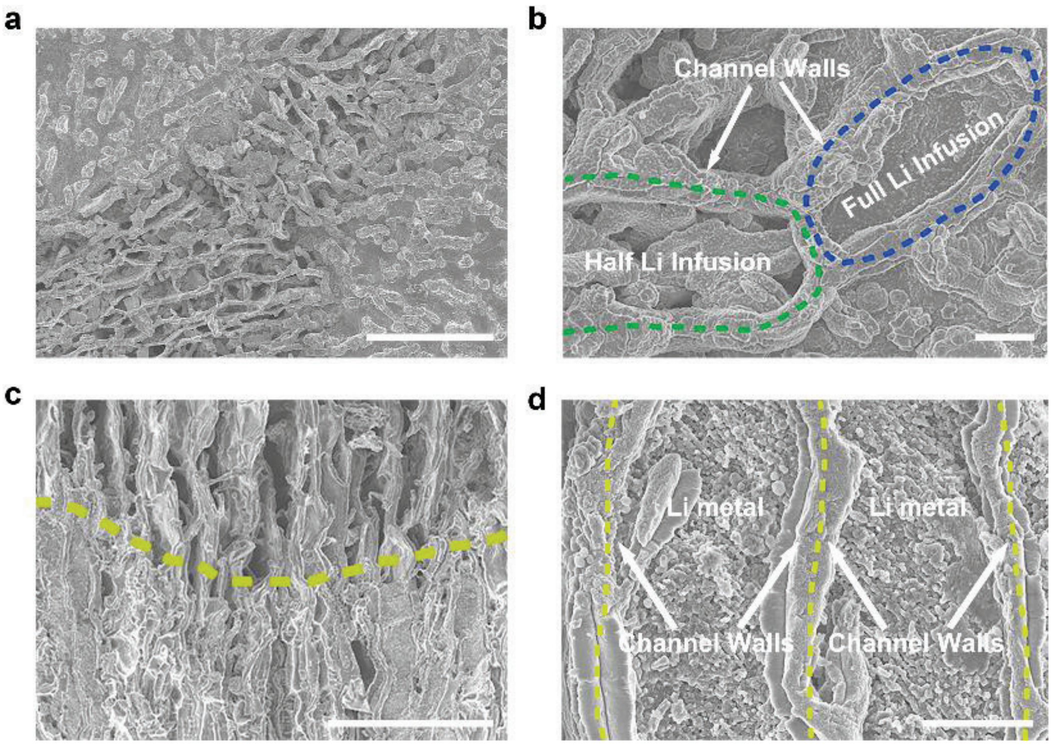

Supplementary Figure 5 | Detailed investigation and characterization of Li infusion into CHVC. (**a**) Top-view SEM image of Li/CHVC composite with half Li infusion. (**b**) Partial enlarged top-view SEM image of Li/CHVC composite, exhibiting the contrast between channels with half Li infusion (encircled by green dotted line) and full Li infusion (encircled by blue dotted line). (**c**) Side-view SEM image of Li/CHVC composite with half Li infusion. (**d**) Partial enlarged SEM image of longitudinal section of Li/CHVC composite, exhibiting the well-preserved vertical channels and Li metal in them after the process of Li infusion.

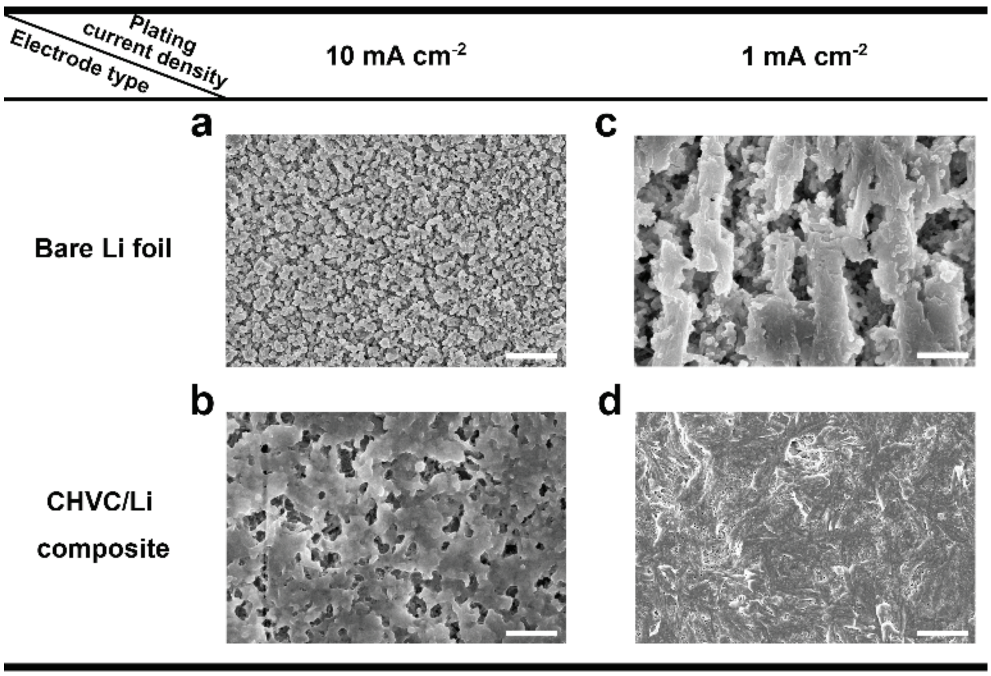

Supplementary Figure 6 | Surface micro-morphologies of different electrodes after Li plating under high (10 mA cm^{-2}) and low current density (1 mA cm^{-2}), respectively. (**a-b**) Surface micro-morphology of bare Li (**a**, scale bar, 5 μm) and CHVC-30/Li composite (**b**, scale bar, 500 nm) after Li plating under 10 mA cm^{-2}, showing the obvious contrast between the loose structure of bare Li surface and relatively dense form of CHVC-30/Li composite surface after high current density Li plating. (**c-d**) Surface micromorphology of bare Li (**c**, scale bar, 500 nm) and CHVC-30/Li composite (**d**, scale bar, 5 μm) after Li plating under 1 mA cm^{-2}, showing the obvious contrast between the nonuniform deposition of bare Li surface and relatively homogeneous deposition of CHVC-30/Li composite surface after low current density Li plating.

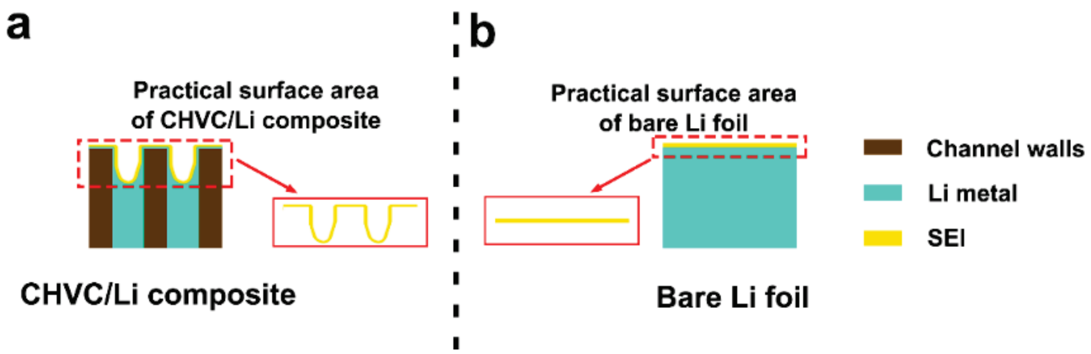

Supplementary Figure 7 | Schematic illustration of mechanism of the excellent performances of CHVC/Li composite electrodes on the basis of enlarged practical surface area. (**a**) Schematic diagram showing the enlargement of the practical surface area of CHVC/Li composite, which can be attributed to the existence of micro-channels, compared to the plat contact surface between Li metal and electrolyte of bare Li foil (**b**).

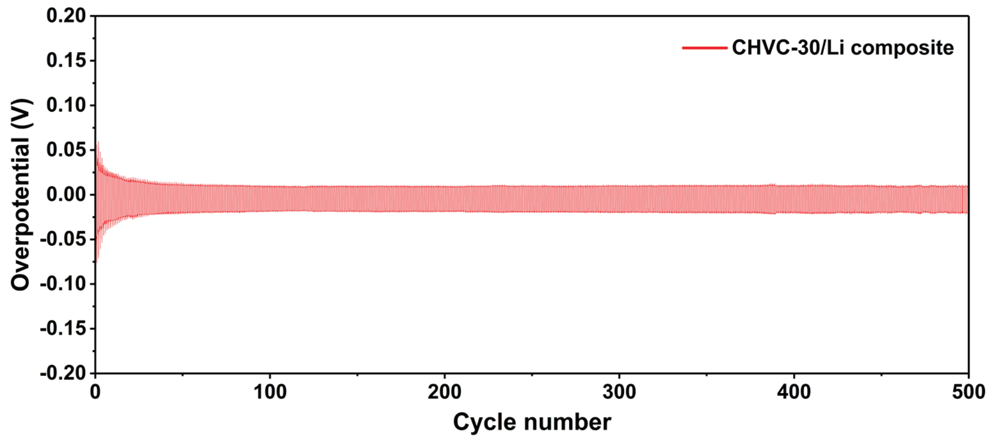

Supplementary Figure 8 | Voltage profile of the symmetric cell with CHVC-30/Li composite electrodes under a current density of 1 mA cm^{-2}, in which CHVC/Li composite electrode exhibits a low overpotential of 18 mV and the cycling stability up to 500 cycles.

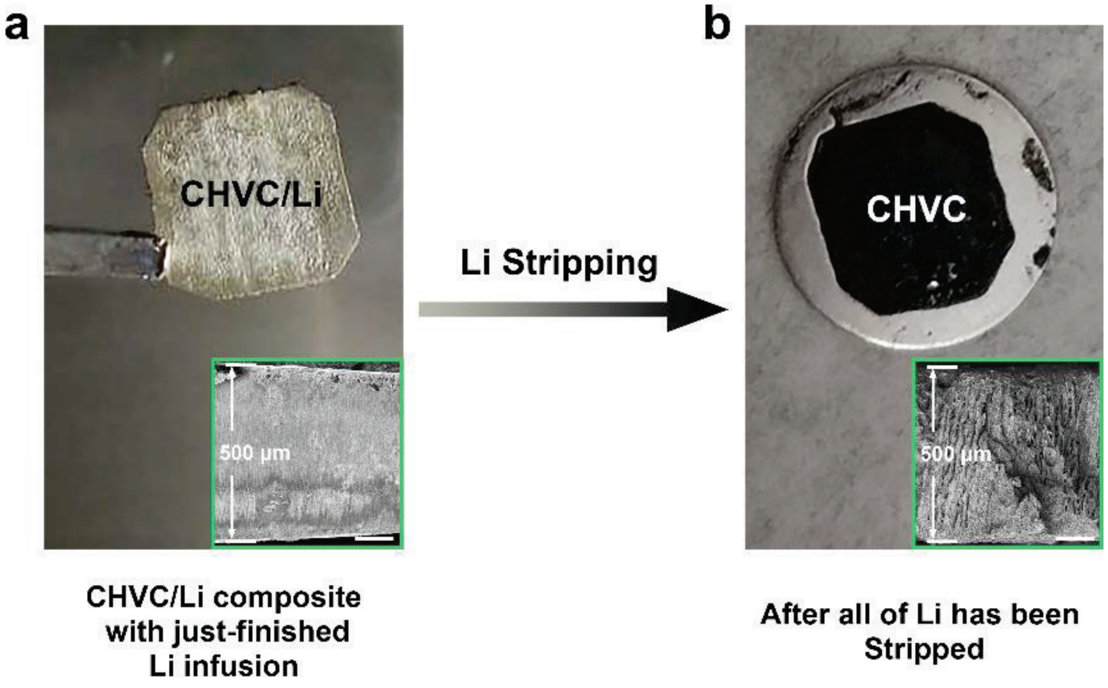

Supplementary Figure 9 | Photos and SEM images of a CHVC/Li composite electrode before and after Li stripping. (**a**) The photo and SEM image of a CHVC/Li composite electrode after just-finished Li infusion process (scale bar, 100 μm). (**b**) The photo and SEM image of a CHVC/Li composite electrode after Li stripping, showing the good preservation of macroscopic shape and microstructure of vertical channels (scale bar, 100 μm).

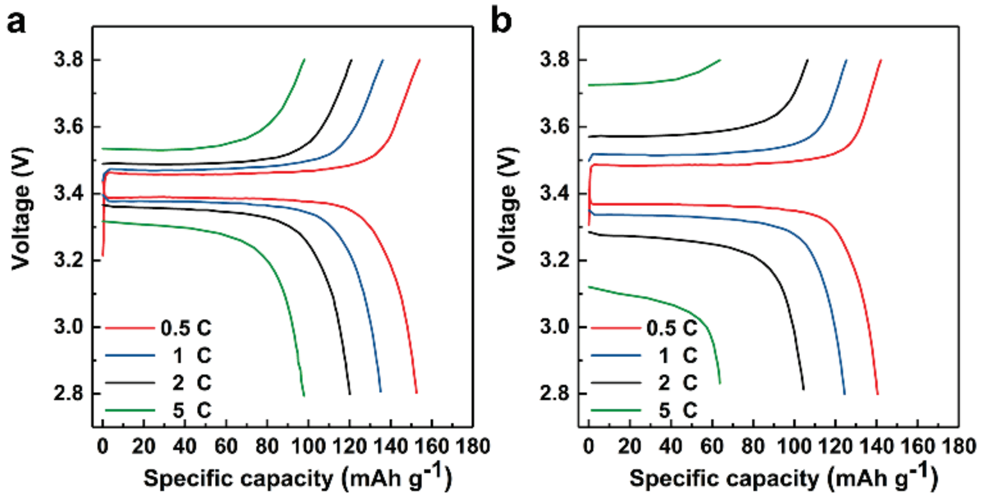

Supplementary Figure 10 | Polarization curves of full cells with CHVC-30/Li composite anode (a) or bare Li anode (b) at different rates.

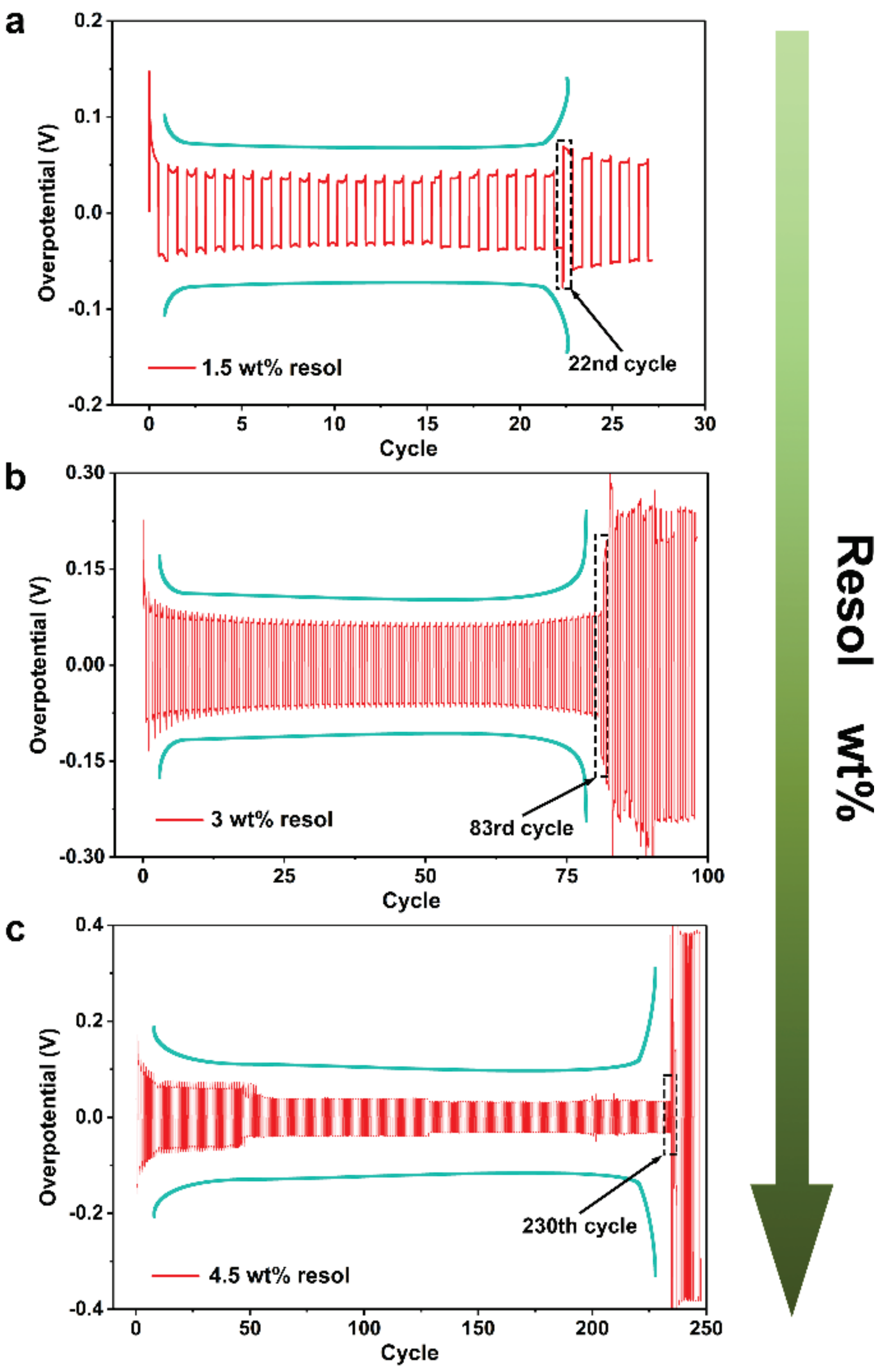

Supplementary Figure 11 | Typical unstable performances during cycling caused by lack of strength of channel walls in CHVCs. The lack of strength can be radically ascribed to inadequate concentration of resol in the solution used to fabricate PMVCs. The typical sudden overpotential increase mentioned in Supplementary Note 1 occurs at 22nd, 83rd, and 230th cycle for CHVCs made of solutions with 1.5 wt% (**a**), 3 wt% (**b**) and 4.5 wt% (**c**) of resol, respectively.

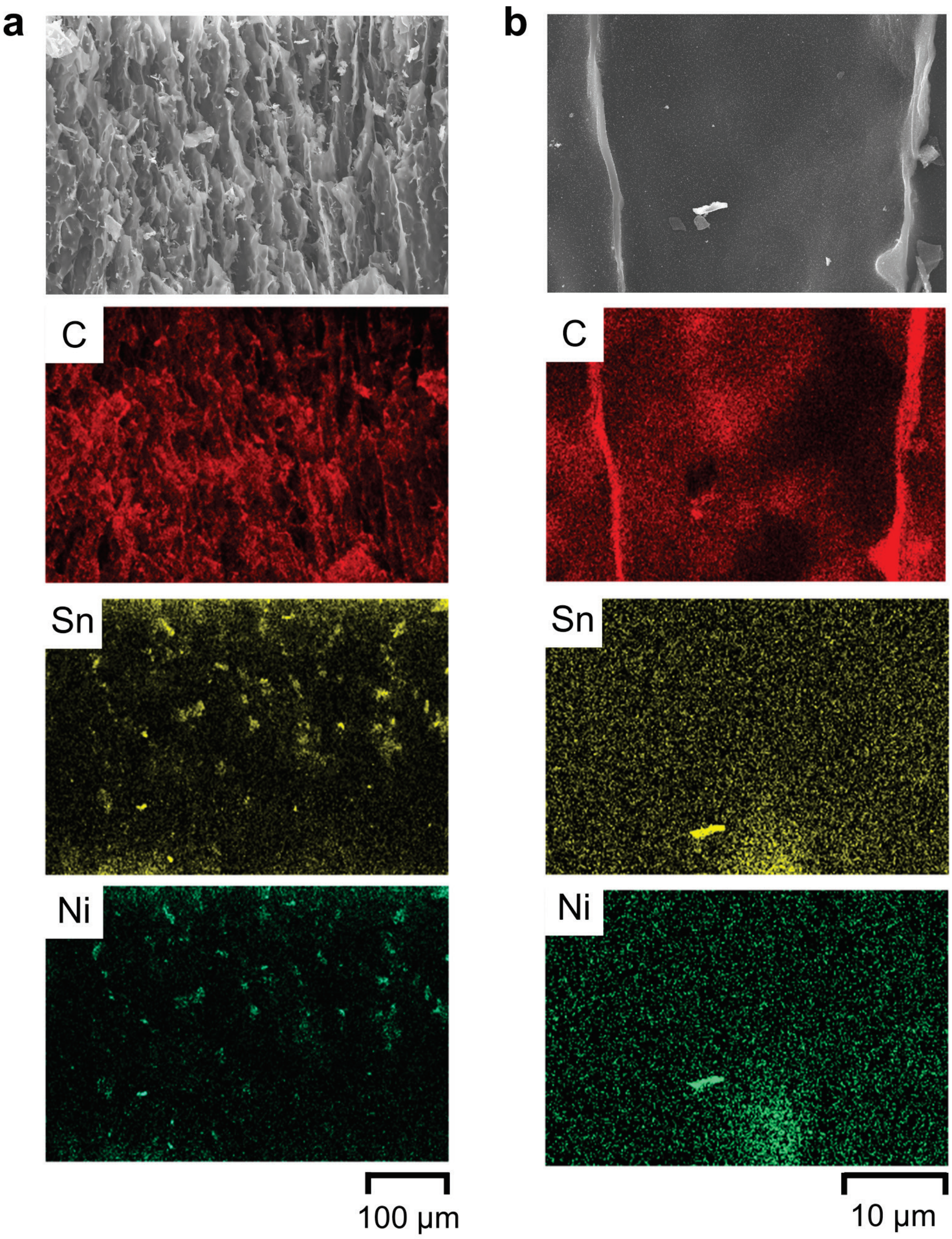

Supplementary Figure 12 | Side-view SEM images with different magnification times and corresponding EDX mappings indicating the dispersion of elements of C, Sn and Ni. Some areas with particularly strong elemental signals of Sn or Ni in Fig. S12 (a) can be attributed to the fragments caused by sample slicing, which leads to those fragments directly facing the signal receiver of EDX and reflecting stronger signals.

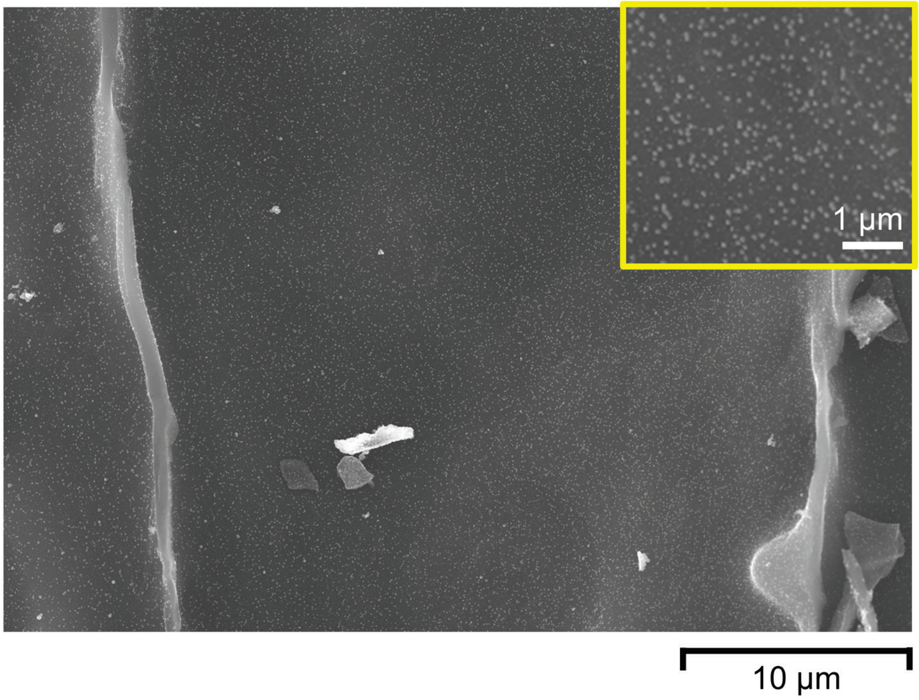

Supplementary Figure 13 | Corresponding images exhibiting more details as a supplement for Fig. S12 (b).

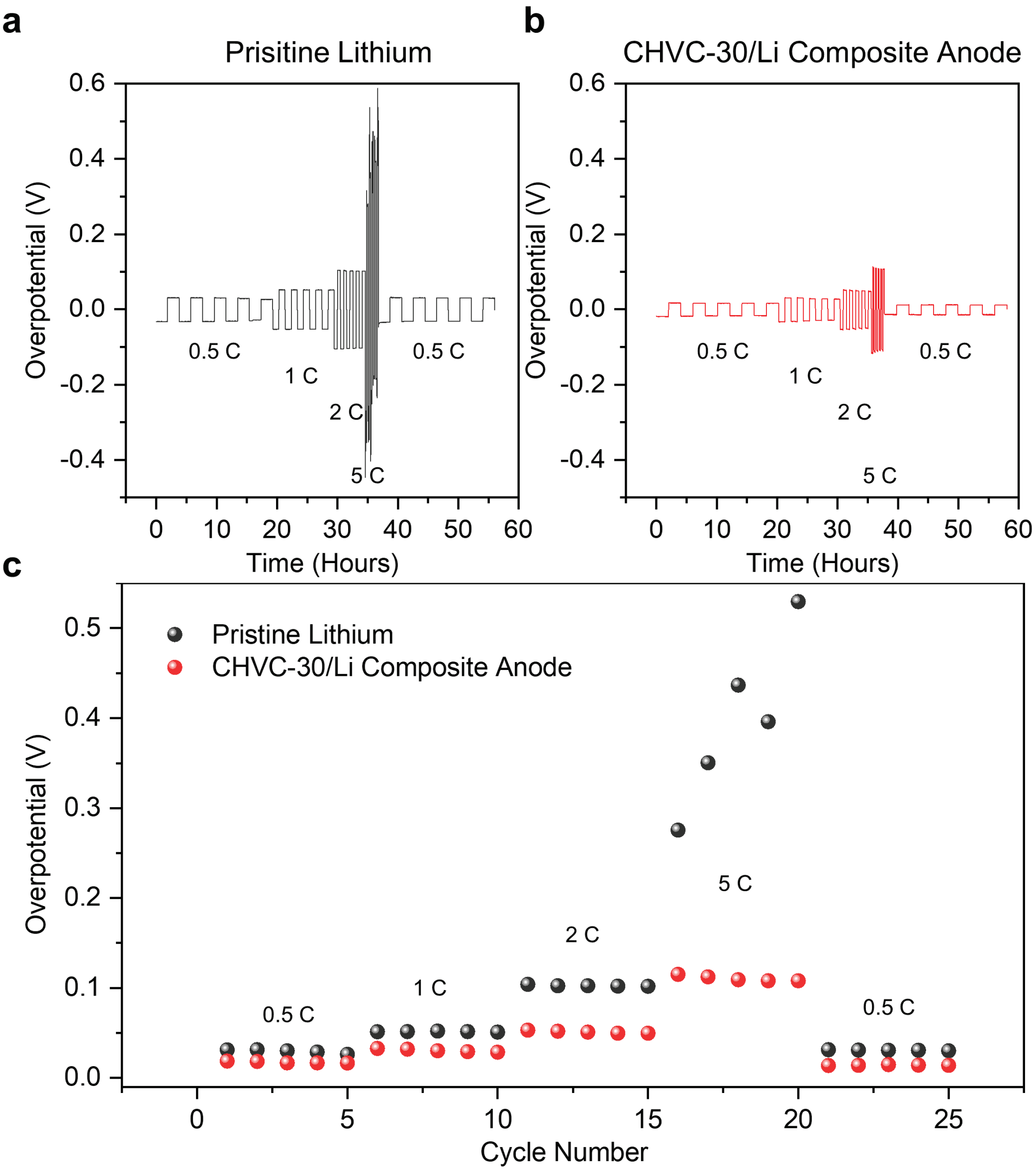

Supplementary Figure 14 | The electrochemical performances of CHVC-30/Li composite anode and pristine lithium at different current densities. (a) The voltage curve of the symmetrical cell with pristine lithium. (b) The voltage curve of the symmetrical cell with CHVC-30/Li composite electrodes. (c) The median voltage values of two cells at different rates. The capacity is set as 2 mAh cm^{-2} and 1 C stands for the current density of 2 mA cm^{-2}.

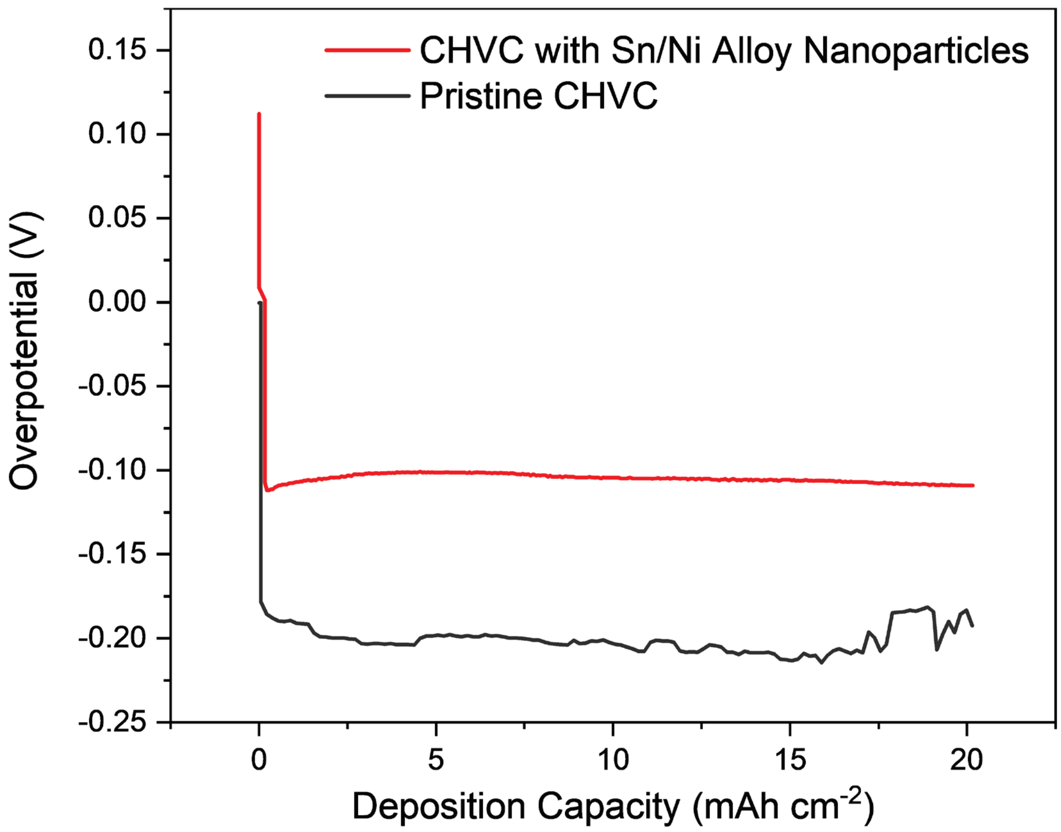

Supplementary Figure 15 | The Plating Curves of CHVC with or without Sn/Ni Alloy Nanoparticles at a current density of 10 mA cm^{-2} and a capacity of 20 mAh cm^{-2}

Supplementary Tables

Supplementary Table 1 | The macroscopic size change of our matrix with vertically aligned channels before (PMVC) and after (CHVC) carbonization and the size change of the micro-channels in them (T_i = - 30 ^oC)

**State of the matrix**	**Macroscopic size (Plan view)**	**Average size of the micro-channels inside**
PMVC	2.11 x 1.82 (cm)	20.2 μm
CHVC	1.49 x 1.24 (cm)	14.1 μm
**Size shrinkage ratio (In one dimension)**	29.4 % ~ 31.9 %	30.2 %

(The statistical method of channels size in PMVC and CHVC is illustrated in **Supplementary Fig. 2**)

Supplementary Table 2 | The cycling performance of full cells with $LiFePO_4$ cathode versus CHVC-30/Li composite or bare Li anode.

Index \ Anode Type	CHVC-30/Li	Bare Li foil
Discharge capacity of the first cycle (mAh g^{-1})	126.5	120.4
Discharge capacity of the 120th cycle (mAh g^{-1})	114.6	98.8
Capacity retention rate	90.6 %	82.1 %

Supplementary Table 3 | The average thickness of micro-channel walls of CHVCs fabricated with solutions with different concentration of resol.

Concentration of resol (wt%)	Average wall thickness of CHVC (μm)	Comments
1.5	0.52	Too thin
3.0	0.91	Too thin
4.5	1.03	Too thin
6.0	2.02	Moderate
7.5	2.83	Too thick

Supplementary Notes

Supplementary Note 1: Composition ratio optimization of the resol-solutions used to fabricate PMVC.

As mentioned in the main text, the solution is mainly composed of resol, chitosan, acetic acid (HAc), graphene oxide (GO) and deionized water. Among them, deionized water is the solvent/dispersant, and GO is added to improve the processability and conductivity of PMVC. Furthermore, chitosan functions as a temporary template of the cryogel[1] and the chitosan will be pyrolyzed under high temperature. The best concentration of chitosan in the solution has been reported in our previous work[1,2].

As the major carbon source of the final CHVC, resol was converted into amorphous carbon with embedded reduced graphene oxide (rGO) after calcination at 900 ^{o}C for 6 hours in the Ar/H_2 atmosphere. Thus the concentration of resol in the solution correlates with the thickness of channel walls of the final products. There is a most appropriate wall thickness of the final CHVC (Supplementary Table 3). The channel walls could resist the volume change during cycling, which means a sufficient strength of the walls is needed to maintain the integrity. Thinner walls may cause collapse of channels during cycling due to the low strength. Excessively thicker walls will occupy too much volume which is desired to accommodate metal Li. To investigate the proper wall thickness, we gradually increased the resol content in the solution (1.5 wt%, 3.0 wt%, 4.5 wt%, 6.0 wt% and 7.5 wt%) and recorded the cycling performances of corresponding CHVC/Li electrodes. When the resol content is lower than 6.0 wt%, the voltage profiles of the final CHVC/Li composite electrodes frequently exhibited a sudden increase after tens of cycles, despite their relatively stable overpotential in the cycling process before (Supplementary Fig. 11). This phenomenon can be attributed to the damages of the micro-channels caused by the

volume changes of Li metal during cycling when these channels are not strong enough. When the concentration reached 6.0 wt% or a larger value, the undesired unstability happened rarely.

Thus, the optimized compositions of the solution were finally adopted as follows: 6.0 wt% resol, 2 wt% chitosan, 2 vol% HAc, and 0.5 wt% GO.

Supplementary Note 2: A brief theoretical explanation for the homogeneity of the size distribution of Ni/Sn nanoparticles electroplated via high-voltage pre-nucleation technology (HVPNT).

The phenomenon can be attributed to two main factors: the higher overpotential accounting for smaller nucleation, and the tip effect.

According to the nucleation theory of Physical Chemistry, in an electro-deposition system, the critical nucleation radius-overpotensial and the nucleation rate-overpotential correlations can be concisely expressed as follows[3,4]:

$$r_c \propto \frac{1}{\eta} \tag{1}$$

$$J = K\exp(-\frac{C}{\eta}) \tag{2}$$

r_c stands for the critical nucleation radius.

η stands for the overpotential between cathode and electroplate liquid.

J stands for the nucleation rate.

K stands for the pre-exponential factor of the system.

C stands for a combination of some factors, the value changes of which can be neglected under non-extreme conditions.

According equation (1) and (2), in an electroplating system, a higher voltage, which means a higher overpotential at the interface between the cathode and the electroplate liquid, leads to a increasing nucleation rate and a smaller critical nucleation radius.

Compared with a plane, surface with a small radius of curvature possesses higher charge density, which leads to the 'tip effect'. Owing to the fairly small size of Ni/Sn crystal nuclei, the tip effect becomes extremely obvious. As a result, when the electroplating program turns into low-current electro-deposition, metal ions in the electroplating liquid tend to choose the position of Ni/Sn

nanoparticles formed in the last step as the place for reduction and deposition.

In conclusion, our electroplating program is composed of two parts. The high-voltage pre-nucleation program mainly provides conditions for the nucleation of Ni/Sn nanoparticles, while the low-current procedure make the nuclei formed in the last step grow to the final size.

Supplementary Note 3: Principles accounting for the better cycling performances of CHVC/Li composite electrode compared with bare Li foil. In virtue of the existence of vertical micro-channels, the practical contact area between metal Li and electrolyte in symmetric cells with CHVC/Li composite electrodes is much larger than that of bare Li symmetric cells (Supplementary Fig. 7). According to the definition, the practical current density equals to the ratio of current to practical surface area. Furthermore, the resistance of a certain segment of an object is inversely proportional to its practical surface area. Noticeably, due to the existence of different phases with different conductivities in a electrochemical system, the approximate calculation of resistance need to be expressed via integration.

$$J_p = \frac{I}{S_p} \tag{3}$$

$$R_t = \int \frac{\rho_l}{S_p} dL \tag{4}$$

J_p stands for the practical current density.

I stands for the value of current flowing through a cell.

S_p stands for the practical surface area.

R_t stands for the total resistance of a cell.

ρ_l stands for the resistivity of a certain segment of a cell.

dL stands for the length of the certain segment.

According to the equation (3), due to a larger practical contact area, the practical current density of CHVC/Li composite electrodes is lower. A lower current density would result in a homogeneous and dense deposition of Li metal during Li plating process, the phenomenon of which is confirmed by SEM images in Supplementary Fig. 6. Furthermore, equation (4) indicates that the larger practical surface area of CHVC/Li composite electrodes leads to a lower resistance, which also means a lower overpotential during cycling. The conclusion of the above inference is in consistence with the electrochemical performances of different cells shown in Fig. 3 of the main body.

Supplementary Movies

Supplementary Movie 1. The infusion of molten lithium into our CHVC framework.

Supplementary References

1 Yu, Z.L. *et al.* Bio-inspired Polymeric Woods. *Sci. Adv.* **4**, eaat7223 (2018).

2 Gao, H. L. *et al.* Mass production of bulk artificial nacre with excellent mechanical properties. *Nat. Commun.* **8**, 287 (2017).

3 Atkins, P. W. & De Paula, J. *Atkins' Physical chemistry*. Tenth edition. edn, (Oxford University Press, 2014).

4 Budevski, E., Staikov, G. & Lorenz, W. J. Electrocrystallization Nucleation and growth phenomena. *Electrochim. Acta* **45**, 2559-2574 (2000).
